# Rheology and 3D Printability of Percolated Graphene–Polyamide-6 Composites

**DOI:** 10.3390/polym12092014

**Published:** 2020-09-03

**Authors:** Kok Peng Marcian Lee, Milan Brandt, Robert Shanks, Fugen Daver

**Affiliations:** 1 School of Engineering, RMIT University, PO Box 71, Bundoora, VIC 3083, Australia; kokpengmarcian.lee@rmit.edu.au; 2 Centre for Additive Manufacturing, RMIT University, Melbourne, VIC 3001, Australia; milan.brandt@rmit.edu.au; 3 School of Science, RMIT University, GPO Box 2476, Melbourne, VIC 3001, Australia; robert.shanks@rmit.edu.au

**Keywords:** graphene, material extrusion, 3D printing, rheological properties, printing envelope

## Abstract

Graphene–polyamide-6 (PA6) composites with up to 17.0%·*w*/*w* graphene content were prepared via melt mixing. Oscillatory rheometry revealed that the dynamic viscoelastic properties of PA6 decreased with the addition of 0.1%·*w*/*w* graphene but increased when the graphene content was increased to 6.0%·*w*/*w* and higher. Further analysis indicated that the rheological percolation threshold was between 6.0 and 10.0%·*w*/*w* graphene. The Carreau–Yasuda model was used to describe the complex viscosity of the materials. Capillary rheometry was applied to assess the steady shear rheology of neat PA6 and the 17.0%·*w*/*w* graphene–PA6 composite. High material viscosity at low shear rates coupled with intense shear-thinning in the composite highlighted the importance of selecting the appropriate rheological characterisation methods, shear rates and rheological models when assessing the 3D printability of percolated graphene–polymer composites for material extrusion (ME). A method to predict the printability of an ME filament feedstock, based on fundamental equations describing material flow through the printer nozzle, in the form of a printing envelope, was developed and verified experimentally. It was found that designing filaments with steady shear viscosities of approximately 15% of the maximum printable viscosity for the desired printing conditions will be advantageous for easy ME processing.

## 1. Introduction

Material extrusion (ME), which encompasses fused filament fabrication (FFF) or fused deposition modelling (FDM) methods, is an additive manufacturing (AM) process that involves extrusion of molten material through a moving nozzle (X–Y plane) onto a moving platform (Z-plane) [1]. While the material feedstock for ME processes can come in various forms, ME will be used to exclusively refer to filament-based methods in this work.

The ME 3D printing methodology is essentially a melt-extrusion process on a much smaller scale, and the rheological characteristics of the material feedstock can be a useful tool in understanding its behaviour during ME processing. Besides melt rheology, several other factors such as filament buckling, local shearing during filament feeding and nozzle clogging can be considered for a complete assessment of material printability. Filament buckling and local shearing can limit the maximum force that can be applied on the filament, thus indirectly affecting the printability of a material [2]. Nozzle clogging can occur due to filament burn in the nozzle or blockages at the nozzle exit [3]. In the case of filled filaments, the presence of fillers can lead to nozzle clogging, and this is a physical limitation that is dependent on the size and volume fraction of the filler relative to the nozzle exit diameter [4]. It has been found that printing speeds have a direct impact on the dimensions of the extruded filament [5]. This can affect the quality of a printed part and therefore impose printing speed as a limitation when assessing materials for printability. While it is recognised that various factors can lead to 3D printing failure, the primary focus of this work is on the rheological criteria for printability assessment.

In its simplest form, matching the melt flow index (MFI) of a novel ME filament material to that of a known suitable material has been applied as an approach towards designing ME filaments. For example, 50%·*w*/*w* aluminium powder was identified as the most suitable filler content for ME of aluminum powder–polyamide-6 (PA6) as it has a similar MFI to a commercial acrylonitrile butadiene styrene (ABS) filament [6]. In a similar approach, the MFI of ABS was set as the minimum MFI that alternative filaments have to be to ensure printability [7]. A general relationship between MFI and filament stiffness was observed when assessing the printability of poly(ethylene-*co*-vinyl acetate) (EVA) [8]. However, the authors were unable to specify boundary values for those parameters.

While not directly determining the printability of a filament, MFI can be applied as a factor in optimisation studies when designing composite ME filaments [9]. A balance between MFI, elastic modulus and tensile strength was used to determine an optimum filler loading in graphene–ABS filaments [10]. Besides mechanical properties, Zhu, et al. [9] considered the relationship between MFI and thermal conductivity when determining the optimum graphene content in graphene–polyamide 12 filaments. Achieving a suitable relative MFI has been listed as one of the main obstacles in ME filament development [11]. Although it has been demonstrated that MFI can indicate material printability, it neither accounts for the influence of shear rate under actual print conditions nor the differences in sensitivity to shear rate when comparing the viscosities of different polymers.

It must be highlighted that the works mentioned above on graphene–polymer composites have not considered filler concentrations above the rheological percolation threshold, as the associated decrease in MFI with increasing graphene concentration was deemed undesirable for printability. The onset of rheological percolation in graphene–polymer composites can impart sudden changes to rheological behaviour, including a significant increase in zero shear viscosity and more intense shear-thinning [12,13,14]. Furthermore, working with graphene concentrations above the percolation threshold is necessary for achieving material properties, such as electrical and thermal conductivities, that depend on the formation of percolated graphene networks. Therefore, assessment of composite printability when graphene concentration is above the rheological percolation threshold is of interest when aiming to print multifunctional components.

The limitations of MFI for determining printability can be overcome by applying rheometric techniques. For example, a target viscosity of between 100–300 Pa·s at printing shear rates was used to verify the printability of graphene–poly(lactic acid) (PLA) and carbon nanotube–PLA composites [15]. In this range of viscosity, the polymer melt was suitable to flow, wet and spread, while supporting itself [16]. In another approach, the identification of a crossover point using dynamic shear rheology was indicative of melt elasticity and was applied to assess the suitability of a material for ME processing [17,18].

The rheological approach to verify printability can be further expanded by modelling material flow in the extruder nozzle. For example, the maximum printable viscosity of a material can be determined by relating the maximum shear stress at the nozzle exit with printing shear rates [19]. The authors demonstrated that the predicted maximum printing speeds for poly(vinyl chloride-*co*-butyl acrylate) in a 3D-bio-plotter agreed well with experimental observations. However, the bio-plotter differed from filament-based ME techniques in terms of the material feed mechanism. Furthermore, the converging cross-section of typical ME nozzles presented an added complexity.

While similar in approach to Calafel, et al. [19], both Duty, et al. [20] and Beran, et al. [4] applied fundamental equations based on volume flow of the material and included considerations specific to the filament-based ME of glass-filled polycarbonate and carbon fiber-reinforced ABS, respectively. However, the authors only verified their approaches at a single print speed with a pass/fail criterion. In a separate study, a theoretical window of optimal extrudability was developed for steel powder– binder composites [21]. The authors assessed the stability of the applied pressure during extrusion and the dimensional variability of the extruded filament to determine the optimal filler content, extrusion temperature and extrusion speed. While the motivation for the study was due to a growing interest in extrusion-based AM, it was not directly applied using AM techniques.

Therefore, we aim to enhance the understanding of rheology of graphene–PA6 composites and their relation to 3D printability as a filament feedstock in ME. The objectives are to (i) investigate the influence of grapheneaddition to the dynamic and steady shear rheology of graphene–PA6 composites; (ii) identify the rheological percolation concentration of graphene–PA6 composites; (iii) develop a concise methodology for predicting the 3D printability of materials, in the form of a printing envelope, as filament feedstock for ME; and (iv) verify the printing envelope experimentally for both neat PA6 and a percolated graphene–PA6 composite.

## 2. Materials and Methods

### 2.1. Materials

PA6 under the trade name ‘Taulman 645’, was supplied by Taulman3D (St. Louis, MO, USA) in the form of a filament. The PA6 was specifically developed for applications in melt extrusion additive manufacturing processes such as fused deposition modelling.

Graphene nanoplatelets (GNP) were supplied by XG Sciences (Lansing, MI, USA). The GNP were specified as “Grade M-25” by the manufacturer, with a typical surface area of 120–150 m^2^/g. They have an average diameter of 25 µm and an average thickness of 6–8 nm.

### 2.2. Composite Preparation via Melt-Mixing

GNP–PA6 composites (GC) with up to 17.0%·*w*/*w* GNP content were prepared. Specimens were labelled GC0.1, GC6.0, GC10.0 and GC17.0, with the numbers representing the weight fractions of GNP in the composite. PA6 and GNP were dried in a vacuum oven at 80 °C for 20 h before compounding. GC in approximately 50 g batches was compounded with a Haake Rheomix 600 Batch Intensive Mixer (Waltham, MA, USA) fitted with counter-rotating roller impellors. Compounding was performed for a total of 15 min at 245 °C and 100 rpm. PA6 was first added to the melt-mixer and allowed to homogenously melt for 5 min. GNP were then added as a dry powder and allowed to disperse for a further 10 min.

### 2.3. Preparation of Test Specimens

Test specimens for rheology studies were prepared as disks of 25 mm diameter and 2 mm thickness by compression molding. Compression molding was performed with a heated press at 245 °C. The material was first allowed to melt at contact pressure for 10 min. The pressure was subsequently ramped to 20 MPa over 1 min, and the pressure was maintained for an additional 5 min. The mold was water-cooled at an average cooling rate of 35 K/min to 60 °C before the test specimen was removed.

The preparation of GC17.0 into a filament for ME was described in our earlier work [22]. ME 3D printing was performed with a MakerBot Replicator 2X (Brooklyn, NY, USA) experimental 3D printer set up with a 0.4 mm diameter nozzle. Specimens measuring 23 × 10 × 0.4 mm were printed with an extruder temperature of 245 °C, print bed temperature of 65 °C, linear infill pattern, 100% infill fraction and a layer height of 0.2 mm. Printing speeds were varied from 1 mm/s to 175 mm/s. PA6 and GC17.0 filaments were dried in a vacuum oven at 80 °C for 20 h before ME processing.

### 2.4. Dynamic Shear Rheology

The dynamic shear rheology of the materials was measured using oscillatory rheometry on a strain-controlled ARES rotational rheometer (TA Instruments) with a force transducer of torque range of 0.2–200 g·cm and 25 mm diameter parallel-plate fixture. A gap of 1 mm between the parallel plates was used for all tests. All measurements were performed at 245 °C in an inert nitrogen environment. Test specimens were left to equilibrate for 15 min before measurement. The linear viscoelastic region was first determined by running a strain sweep test between the strain range of 0.065–270% at an angular frequency (ω) of 1 rad/s. Subsequently, frequency sweep tests were conducted on fresh test specimens between the ω range of 0.1–100 rad/s at a strain of 0.1%. Test specimens were dried in a vacuum oven at 80 °C for 20 h before the test.

### 2.5. Steady Shear Rheology

Steady shear rheology of PA6 and GC17.0 was assessed using an CEAST SR20 capillary rheometer (Norwood, MA, USA) with a double bore configuration. The capillary rheometer has a maximum force range of 20 kN and bore diameter of 15 mm. Steady shear rheology tests were conducted at 245 °C with data collected at the shear rates ($\;\dot{\gamma }$) of 10, 100, 500, 1000, 2000, 4000 and 8000 s^−1^. Dies with Length/Diameter ratios of 20 and 80 were used for each specimen. Both Bagley and Rabinowitsch corrections were performed [23]. Test specimens were dried in a vacuum oven at 80 °C for 20 h before the test.

## 3. Theory and Calculations

### 3.1. Carreau–Yasuda Model

The Carreau–Yasuda model is defined by Equation (1) [24]:

(1) $$\eta^{*}\left(\omega \right)\;={\;\eta }_{0}^{*}{\left[1+{\left(\lambda \omega \right)}^{\alpha }\right]}^{\frac{n-1}{\alpha }}$$

where η* is the complex shear viscosity and η_0_* is the complex zero-shear viscosity. λ is the characteristic time, n refers to the power-law index and α is indicates the width of the transition region between Newtonian and power-law behaviour. λ and α were obtained by fitting experimental data to the Carreau–Yasuda model for the highest coefficient of determination (R^2^) values. The η_0_* was determined from low shear-rate viscosity data.

### 3.2. Printing Envelope

In this work, nozzle clogging was deemed unlikely and was not considered as the equivalent volume fraction of GNP in GC17.0 was low at approximately 10%, and the geometric ratio of nozzle diameter to filler diameter was high at a ratio of 16 [4].

**Figure 1:**
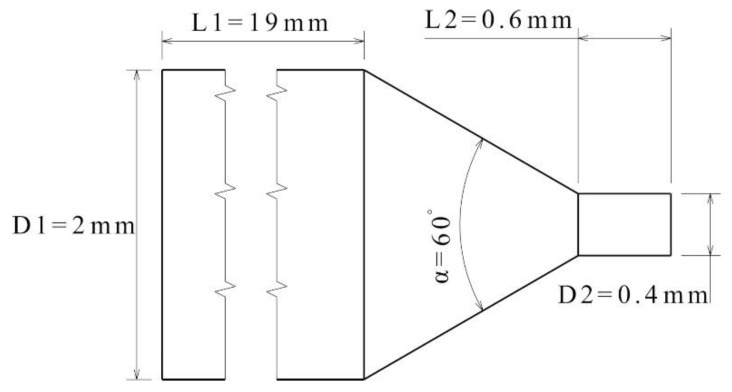
Cross-section of the nozzle and the relevant dimensions.

The flow at the nozzle exit is considered the most critical for predicting the printability of the filament. The cross-section of the nozzle and associated dimensions are presented in Figure 1.

A correction factor (*c*) accounting for the variance in pressure drop across the nozzle due to its converging cross-section was calculated using Equation (2) [4]:

(2) $$c\;=\;\frac{{\Delta P}_{\mathrm{tip}}}{{\Delta P}_{\mathrm{total}}}\;=\;\frac{\frac{L_{2}}{{\left(\frac{D_{2}}{2}\right)}^{\frac{\left(m+3\right)}{m}}}}{\left(\frac{L_{1}}{{\left(\frac{D_{1}}{2}\right)}^{\frac{\left(m+3\right)}{m}}}+\;\frac{m}{3\;\tan\left(\frac{\alpha }{2}\right)\;}{\left(\frac{8}{D_{2}^{3}}-\;\frac{8}{D_{1}^{3}}\right)}^{\frac{1}{m}}+\;\frac{L_{2}}{{\left(\frac{D_{2}}{2}\right)}^{\frac{\left(m+3\right)}{m}}}\right)}$$

where D and L refer to the respective diameter and length of the cylindrical sections in the nozzle, α is the convergence angle, and m = 1/n. ${\Delta P}_{\mathrm{tip}}$ and ${\Delta P}_{\mathrm{total}}$ refer to the pressure drop across the nozzle tip (section D2, L2) and the total pressure drop, respectively. It is assumed that the density of the filament remains constant and the nozzle is surrounded by ambient pressure.

The pressure drop across the nozzle tip is then calculated by Equation (3) [4]:

(3) $${\Delta P}_{\mathrm{tip}}\;=\frac{4\mathrm{cF}}{{\pi D}_{\mathrm{fil}}^{2}}$$

where F refers to the force applied by the 3D printer, and D_fil_ refers to the filament diameter. As the maximum force decreases with increasing printing speed, 40 N was applied as a conservative estimate [2].

Maximum shear stress (σ) at the nozzle wall can then be determined by Equation (4):

(4) $$\sigma \;={\;\Delta P}_{\mathrm{tip}}\frac{D_{2}}{4L_{2}}$$

The shear rate, $\dot{\gamma }$, at the nozzle wall can be calculated by Equation (5) [19]:

(5) $$\dot{\gamma }=\;\frac{3n+1}{4n}\cdot \frac{8v}{D_{2}}$$

For successful printing, the exit velocity at the nozzle must be equivalent to the printing speed. Therefore, v refers to the printing speed.

Shear viscosity (η) at the specific $\dot{\gamma }$ can then be determined by Equation (6):

(6) $$\eta =\frac{\sigma }{\dot{\gamma }}$$

Therefore, by combining Equations (3)–(6), the maximum material η for a specific print speed is given by Equation (7):

(7) $$\eta \;=\;\frac{4n}{3n+1}\cdot \frac{{\mathrm{cFD}}_{2}^{2}}{8{\pi D}_{\mathrm{fil}}^{2}L_{2}v}$$

## 4. Results and Discussion

### 4.1. Dynamic Shear Rheology

**Figure 2:**
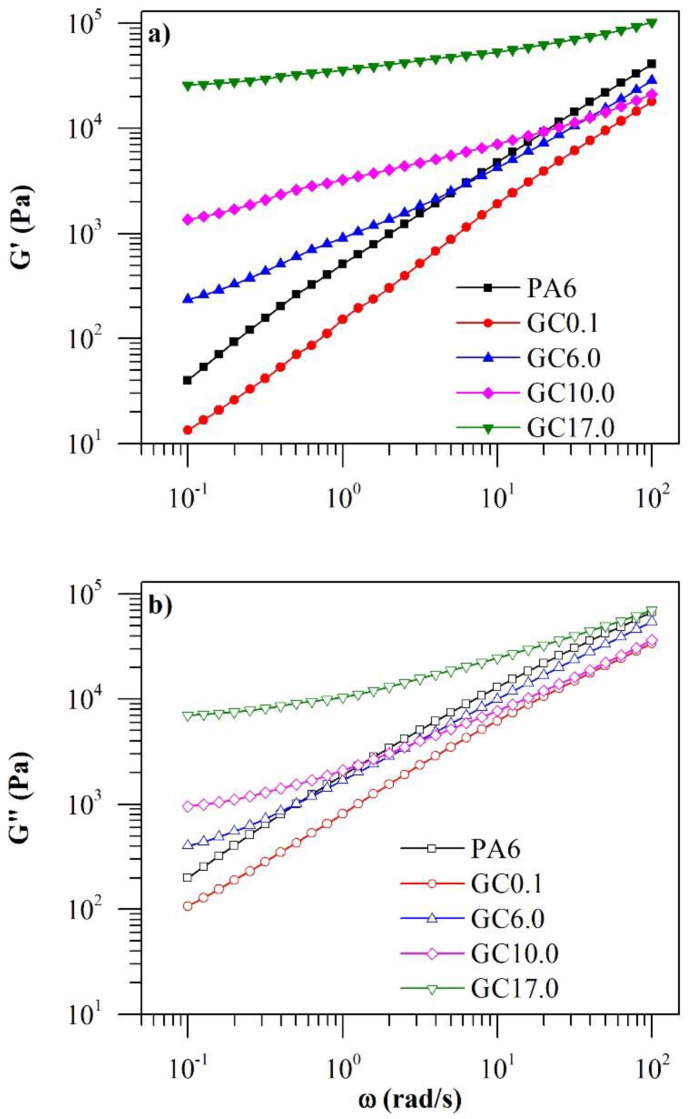
Change in (**a**) G’ and (**b**) G” of neat PA6 and GC with a change in ω at 245 °C.

Dynamic shear rheology was applied as a means to investigate the fundamental flow behaviour of the materials to gain insight into their structure and processability. The ω dependency of the dynamic storage and loss moduli (G’ and G”, respectively) is depicted in Figure 2.

At a low GNP content of 0.1%·*w*/*w*, the addition of GNP resulted in a decrease in G’ and G” when compared to neat PA6. In our previous work on low-defect graphene–PA6 composites, we found that the quality of the filler–matrix interface was best when the filler concentration was low at 0.1%·*w*/*w* [25]. Liu, et al. [26] reported that a decrease in moduli was similarly observed in ultrahigh-molecular-weight polyethylene (UHMWPE) containing low concentrations of reduced graphene oxide. The observation was found to be dependent on the adhesion between graphene and the polymer chains. Therefore, the decrease in moduli with the addition of low concentrations of graphene in this work can be attributed to inhibited polymer chain mobility as a result of good graphene dispersion and strong filler–matrix interactions [26].

However, a subsequent increase in G’ and G” in the low-frequency region was observed when the GNP content was increased to 6.0%·*w*/*w* and higher. The change in trend suggests a change in the graphene dispersion state, such as GNP aggregation as a result of the higher filler content [27]. Between neat PA6 and GC17.0, there was an increase in G’ by three orders of magnitude with GNP addition, while G” only increased by one order of magnitude. The greater increase in G’ can be attributed to the filler’s more pronounced influence on the elastic response of PA6 than its viscous response, thus the materials were becoming more solid-like (where G’ > G”). Increasing moduli with increasing graphene concentration has been widely reported in several graphene–polymer composites including those with PA6 [12], PLA [28] and poly (vinylidene fluoride) (PVDF) [29] matrices. In the mentioned works, graphene concentrations of as low as 0.5%·*w*/*w* were studied. The reduction in moduli observed at low concentrations of reduced graphene oxide in UHMWPE was found to not persist at higher graphene concentrations [26]. In the context of this work, the literature therefore indicates that a general increase in moduli with increasing graphene concentration can be expected when the GNP concentration is between 0.1 and 6.0%·*w*/*w*.

Regardless of the GNP content, all specimens displayed increasing G’ and G” with increasing angular frequency. However, the frequency dependence of G’ and G” had diminished with increasing GNP content. Furthermore, the moduli of specimens with GNP content of 6.0%·*w*/*w* and higher appeared to approach a plateau at low frequencies, with GC17.0 displaying the most distinct plateau. Decreasing frequency dependence and the appearance of a plateau at low frequencies can be attributed to the increasing influence of interactions between adjacent GNP particles as a result of the formation of interconnected structures [28]. Thus, the observations indicate the formation of a percolated network of GNP at concentrations of 6.0%·*w*/*w* and higher.

**Figure 3:**
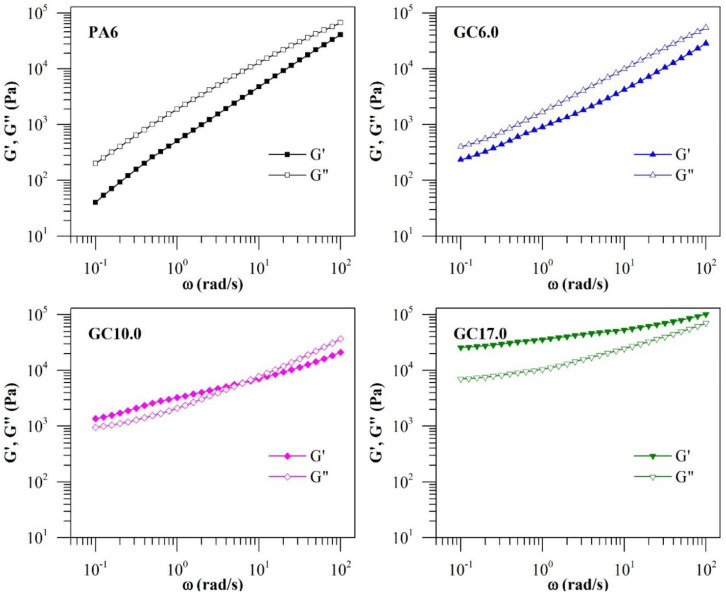
Comparison between G’ and G” of neat PA6 and GC.

The G’ values of the materials are compared with their respective G” in Figure 3. It was observed that G’ was greater than G” across the entire angular frequency range for neat PA6, GC0.1 (not shown in the figure) and GC6.0. A single crossover point could be identified in GC10.0 at the ω of ~7 rad/s. In GC17.0, G’ was higher than G” across the entire angular frequency range. The observation indicates a transition from a liquid-like rheological behaviour to a more solid-like behaviour at GNP concentrations higher than 6.0%·*w*/*w*. The observation of a crossover also indicates that a rheological percolation region occurs between 6.0 and 10.0%·*w*/*w* GNP concentration [14].

**Figure 4:**
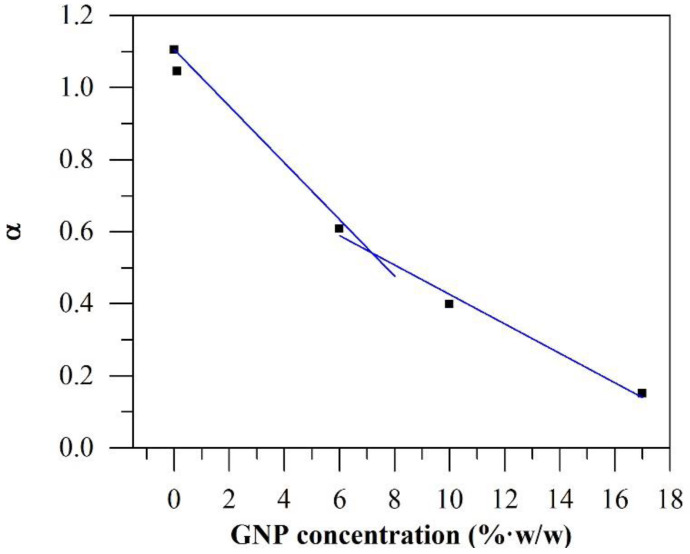
Change in α with increasing GNP content.

**Figure 5:**
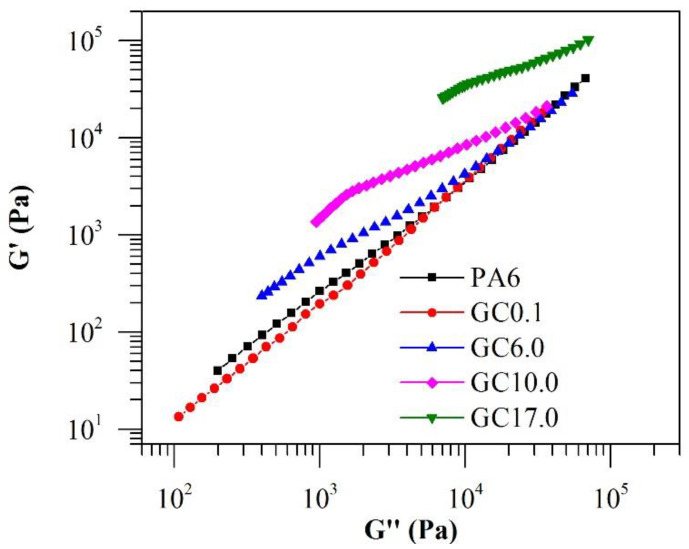
Cole-Cole plots of log G’–log G” for PA6 and GC.

The percolation threshold refers to the critical concentration at which a 3-dimensional percolating network of GNP forms and filler-filler interactions become significant. The formation of percolated GNP networks can manifest as changes in viscoelastic behaviour due to the network’s restrictions on polymer chain mobility [12]. Besides the observation of moduli crossover, rheological percolation can be observed in the change in the slope (α) of the log G’–log ω plot in the terminal frequency region [28]. A change in behaviour of α will correspond to a change from liquid-like to solid-like behaviour. It was observed that the addition of 0.1%·*w*/*w* GNP had a decreasing effect on the α value of PA6 (Figure 4). Thus, neat PA6 became increasingly solid-like despite the small concentration of GNP. With increasing GNP content, α decreased to a minimum of 0.15 for GC17.0. It appears that the influence of GNP addition on α relative to its concentration diminished at higher GNP concentrations with no clear transition from liquid-like to solid-like behaviour. However, the reduced contribution of GNP concentration to α (as evidenced by the change in slope in Figure 4) suggests that the percolation was achieved between 6.0 and 10.0%·*w*/*w* GNP. Further evidence of percolation can be observed from a Cole-Cole plot of log G’–log G” (Figure 5). While the curves for neat PA6 and GC0.1 were linear in the plot, deviations from the linear relationship between G’ and G” were apparent in composites with GNP concentration of 6.0%·*w*/*w* and higher. As such, the results conclusively indicate that the rheological percolation threshold of GC lies between 6.0 and 10.0%·*w*/*w* GNP.

**Figure 6:**
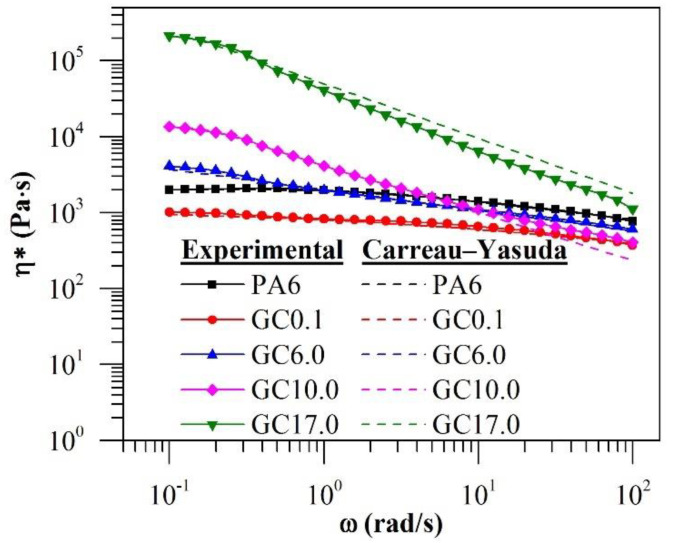
The η* of PA6 and GC with a change in ω at 245 °C.

The complex viscosity (η*) of the materials with respect to the ω is depicted in Figure 6. Neat PA6 exhibited a Newtonian plateau at low frequencies, with slight shear-thinning behaviour at higher frequencies. The addition of 0.1%·*w*/*w* GNP decreased the η* and reduced the extent of the Newtonian region when compared with neat PA6. Increasing the GNP content to 6%·*w*/*w* and higher increased the η* at low frequencies, with more significant shear-thinning behaviour at higher frequencies and the disappearance of the Newtonian plateau in the low-frequency region. The increase in the η* at low frequencies can be ascribed to impeded polymer chain mobility as a result of increased filler-matrix interactions when the GNP concentration increased [28]. The disappearance of the Newtonian plateau is indicative of the formation of continuous GNP networks [30]. It was observed that the η* of GC10.0 was lower than that of neat PA6 and almost equivalent to that of GC0.1, with GC17.0 approaching that of neat PA6 at high angular frequencies as a result of more intense shear-thinning in GC10.0 and GC17.0.

The η* of the materials was further evaluated by fitting the experimental results to the Carreau–Yasuda model (Figure 6: dashed line). The fitted parameters (Equation (1)) and R^2^ of the respective curves are presented in Table 1. Based on the high R^2^, the Carreau–Yasuda model was found to be in good agreement with the experimental results. It was found that the addition of GNP generally increased the characteristic time (λ) when compared with neat PA6. As 1/λ represents the critical shear rate at which shear-thinning behaviour begins, the results indicate that GNP addition induced an earlier onset of shear-thinning when compared with the neat polymer. However, it appeared that the λ was maximum in GC6.0 and subsequently decreased with increasing GNP content. Similar observations have been reported when the viscosity curves of graphene–PVDF composites were fitted to the Carreau model, although the authors did not explain the decrease in λ at high graphene concentrations [31]. It was found that n decreases with increasing GNP content, indicating more intense shear-thinning. The decrease in n was observed to be more pronounced when the GNP content was greater than 6.0%·*w*/*w*.

The observations were found to correlate with the rheological percolation threshold. At a GNP content below that for rheological percolation (<6.0%·*w*/*w*), the addition of GNP had a greater effect on the extent of the Newtonian region. This is because, at a low GNP content, composite rheology is mainly influenced by polymer-to-polymer and particle-to-polymer interactions [32]. An increasing GNP content leads to increased polymer chain entanglements, which subsequently manifests as a shift in the onset of shear-thinning to a lower shear rate (increasing λ) [33]. Overall shear-thinning behaviour depended on the shear-thinning of the polymer chains with some modification by the presence of GNP, and therefore the change in n was small.

At GNP concentration above that of the rheological percolation threshold, particle-to-particle interactions become more dominant and the breakage of the GNP networks at high frequencies will intensify the shear-thinning behaviour of the composites [32]. The shear-induced alignment of the GNP particles can also contribute to the shear-thinning behaviour in the composites [34]. The change in the state of GNP dispersion may have impaired polymer chain entanglement, thus the reduced λ observed in GC10.0 and GC17.0.

The trend in the η* of GNP–PA6 composites differed from that reported by Mayoral, et al. [12], where the η* of neat PA6 remained lower for all composites of between 5 to 20%·*w*/*w* graphene concentration and frequency of up to 100 rad/s. The differences can be attributed to the differences in the graphene used. Firstly, the graphene used by Mayoral, et al. [12] had a diameter of 5 µm, while the GNP diameter was five times as large at 25 µm. As a result, the rheological percolation threshold of the graphene–PA6 system in Mayoral, et al. [12] was greater at 10–15%·*w*/*w*. Considering that shear-thinning behaviour in graphene–polymer composites is intensified by the presence of percolated graphene networks, more intense shear-thinning behaviour was observed at lower graphene concentrations in this work.

### 4.2. Steady Shear Rheology and Printing Envelope

**Figure 7:**
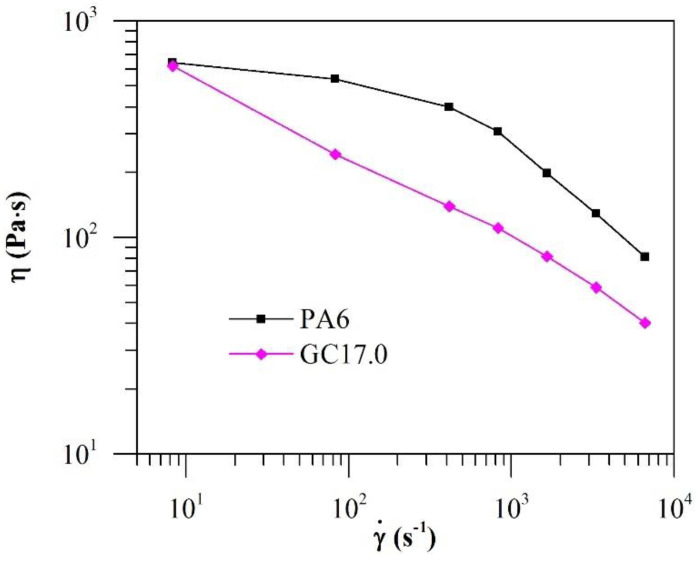
The η of PA6 and GC17.0 with change in $\dot{\gamma }$ at 245 °C.

While dynamic shear rheology provides fundamental knowledge on the microstructure and state of the filler network in the materials, steady shear rheology can provide key information on the processability of the material. Capillary rheometry was applied to evaluate the steady shear rheology of PA6 and GC17.0 at high shear rates that are characteristic of ME printing conditions (Figure 7).

It was observed that at the $\dot{\gamma }$ of 10 s^−1^, both PA6 and GC17.0 had similar η. Although some shear-thinning was observed in PA6 at the $\dot{\gamma }$ of 10 to 100 s^−1^, it was not as intense as that observed in GC17.0. However, at higher $\dot{\gamma }$, more intense shear-thinning was observed in PA6. The n of PA6 and GC17.0 was determined to be 0.47 and 0.58, respectively, for the $\dot{\gamma }$ of between 500 and 8000 s^−1^. The results at a low shear rate agree with the melt flow index (MFI) of the materials, where we reported similar MFI in PA6 and GC17.0 [22]. However, the high shear rate behaviour of the materials was not captured by MFI testing. As demonstrated in our results, relying on MFI for prediction of material printability is insufficient as the shear rates experienced by materials during ME printing are typically high.

**Figure 8:**
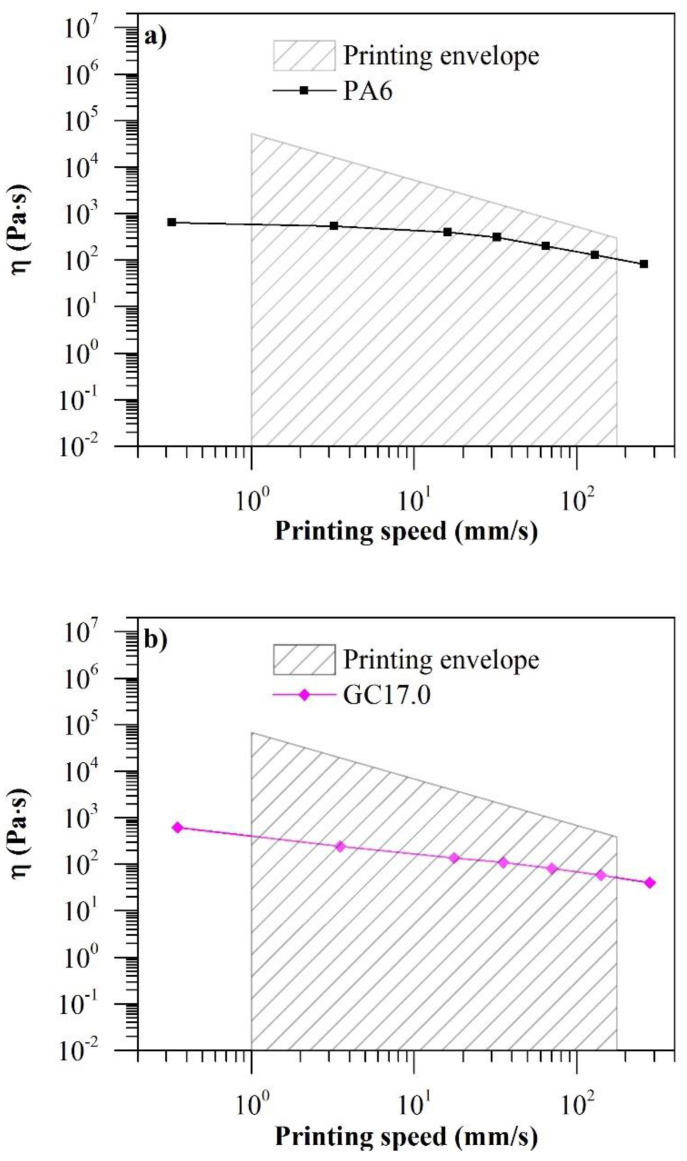
Printing envelope of (**a**) PA6 and (**b**) GC17.0 for printing speeds between 1 and 175 mm/s.

A printing envelope based on the steady shear rheological properties of PA6 and GC17.0 was developed to determine the printability of the materials via ME. The experimental material η overlaid on their predicted printing envelopes is presented in Figure 8. The maximum material η for their respective printing speeds was determined by Equation (7). From the calculated maximum material η, the range of material η that makes it printable at the corresponding printing speeds is visualised as an envelope (Figure 8: shaded region). It was predicted that both neat PA6 and GC17.0 were printable across the entire range of speeds.

**Figure 9:**
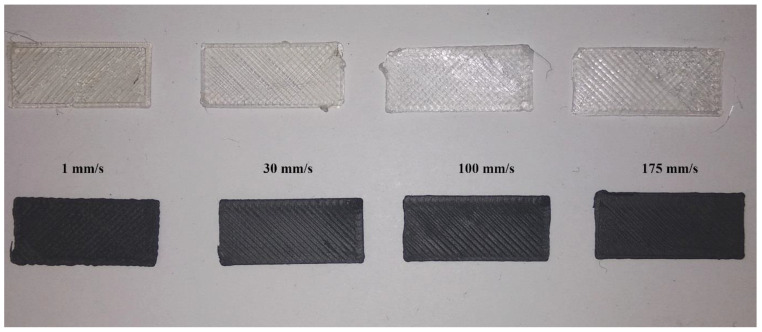
Printed PA6 (**clear**) and GC17.0 (**black**) at various printing speeds.

The printing envelope was verified experimentally (Figure 9). As predicted, both PA6 and GC17.0 were successfully printed at speeds between 1 and 175 mm/s. PA6 could be printed without further adjustments at print speeds of 1 and 30 mm/s. However, a rough surface texture could be observed in PA6 printed at 1 mm/s, indicating over-extrusion. A smooth surface texture was achieved when PA6 was printed at 30 mm/s. While it could still be extruded through the nozzle, PA6 could not stick to the build platform when print speeds were increased to 100 and 175 mm/s. This was attributed to under-extrusion of the filament as the experimental η of PA6 was closer to the predicted limit at the printing speeds. As internal calculations for the volume flow of material by the software were calibrated for ABS, the software may have underestimated the required volume flow of PA6. By creating a custom print setting with adjustments to the “filament diameter” and “feedstock multiplier” parameters, the volume flow of PA6 through the nozzle was increased, and the test specimens were then printed successfully. Although the surface quality of PA6 printed at 100 and 175 mm/s was poor, this was caused by software settings not being optimized and not a physical limitation. In GC17.0, over-extrusion could be observed at low printing speeds. However, this was primarily alleviated when the printing speed was increased to 175 mm/s. Therefore, from a rheological point of view, the printability of both PA6 and GC17.0 from 1 to 175 mm/s has been verified.

The relationship between the measured η of the materials, their respective maximum printable η and surface quality was investigated. It was observed that at 1 mm/s, the measured η of both PA6 and GC17.0 was ~1% of the maximum η. A good surface texture was observed when the material η was ~18% (PA6, 30 mm/s) and ~14% (GC17.0, 175 mm/s) of the maximum printable η. In PA6, when the material η was >30% (100 mm/s and 175 mm/s) of the maximum printable η, under-extrusion was observed. Therefore, it may be advantageous to design ME filaments to have a η of approximately 15% of the maximum printable η at the desired printing conditions for ease of ME processing. It should, however, be noted that these observations are unique to specimens printed on the MakerBot Replicator 2x and using the MakerBot Desktop software (32-bit version 3.10.1.1746, Brooklyn, NY, USA). More advanced software or ME printers may be able to fine-tune the volume flow of the material better when printing at different speeds.

## 5. Conclusions

GNP-PA6 composites with up to 17.0%·*w*/*w* GNP concentration were prepared via melt mixing. The addition of GNP significantly altered the dynamic and steady shear behaviour of PA6. At a low GNP concentration of 0.1%·*w*/*w*, the viscoelastic properties (G’, G” and η*) of PA6 were reduced. However, increasing the GNP concentration to 6.0%·*w*/*w* resulted in an overall enhancement to the properties as mentioned above, especially in the low-frequency region. Observations of crossover in GC10.0, changes in α trends with increasing GNP content and deviation from linear behaviour in the Cole-Cole plot indicate that the critical GNP concentration for rheological percolation was between 6 and 10%·*w*/*w*. The Carreau–Yasuda model was used to describe the η* of the materials. It was observed that increasing the GNP content resulted in more intense shear-thinning and a decrease in the extent of the Newtonian region when compared with neat PA6.

Capillary rheometry revealed that at low $\dot{\gamma }$ of 10 s^−1^, the η of PA6 and GC17.0 was similar. However, GC17.0 was less viscous than neat PA6 when 10 < $\dot{\gamma }$ < 8000 s^−1^. This was despite a two-decade increase in the η* over PA6 when the frequency was low. Therefore, shear-thinning must be accounted for in rheology-based printability modelling for percolated graphene–polymer composites such as GC17.0; otherwise, the model becomes too conservative.

A concise rheological approach for predicting the printing envelope of filament-based ME materials was developed. The approach was experimentally verified by printing PA6 and GC17.0 at various printing speeds. It was observed that the predicted printing envelope was in good agreement with the observed printing results. Observations of under- and over-extrusion were found to be related to the difference between the theoretical maximum printable η and the actual material η. The applicability of the model for both neat PA6 and the percolated graphene–PA6 composite was demonstrated. It is recommended that the target η of the filament feedstock be approximately 15% of the maximum printable η under the desired printing conditions for ease of ME processing.

## Figures and Tables

**Table 1:** Carreau–Yasuda model parameters of PA6 and GC.

Specimen	η_0_* (Pa∙s)	α	λ (s)	n	R^2^
PA6	2013	1.9	0.49	0.77	0.99
GC0.1	1022	0.5	0.46	0.77	0.99
GC6.0	4097	5.6	14.1	0.73	0.99
GC10.0	13550	4.8	6.2	0.37	0.99
GC17.0	213729	8.7	7.5	0.28	0.99
